# NSiteMatch: Prediction of Binding Sites of Nucleotides by Identifying the Structure Similarity of Local Surface Patches

**DOI:** 10.1155/2017/5471607

**Published:** 2017-07-25

**Authors:** Jie Sun, Ke Chen

**Affiliations:** School of Computer Science and Software Engineering, Tianjin Polytechnic University, Tianjin 300387, China

## Abstract

Nucleotides play a central role in life-form metabolism, by interacting with proteins and mediating the function of proteins. It is estimated that nucleotides constitute about 15% of the biologically relevant ligands included in PDB. Prediction of binding sites of nucleotides is useful in understanding the function of proteins and can facilitate the in silico design of drugs. In this study, we propose a nucleotide-binding site predictor, namely, NSiteMatch. The NSiteMatch algorithm integrates three different strategies: geometrical analysis, energy calculation, and template comparison. Unlike a traditional template-based predictor, which identifies global similarity between target structure and template, NSiteMatch concerns the local similarity between a surface patch of the target protein and the binding sites of template. To this end, NSiteMatch identifies more templates than traditional template-based predictors. The NSiteMatch predictor is compared with three representative methods, Findsite, Q-SiteFinder, and MetaPocket. An extensive evaluation demonstrates that NSiteMatch achieves higher success rates than Findsite, Q-SiteFinder, and MetaPocket, in prediction of binding sites of ATP, ADP, and AMP.

## 1. Introduction

Nucleotides are a group of small chemicals that serve as the monomer unit for forming DNA and RNA. They are also the source of chemical energy which is indispensable for majority of the cellular activities. Therefore, nucleotides play a central role in life-form metabolism, by interacting with proteins and mediating the function of proteins. It is estimated that nucleotides constitute about 15% of the biologically relevant ligands included in PDB (Dessailly et al., 2008; Shin and Cho, 2005). In the past, substantial efforts were expended in the identification and characterization of the nucleotide-binding sites. Most of these approaches analyzed the known nucleotide-binding protein sequences and structures to identify conserved motifs. For instance, the Walker A sequence motif was found in a variety of nucleotide-binding proteins that include the alpha and beta subunits of ATP synthase, myosin, transducin, helicases, kinases, and RecA [1]. Moodie and colleagues proposed a fuzzy recognition template for the characterization of the adenylate-protein interactions [2]. However, the abovementioned studies characterize the sequence and structural motifs for a relatively narrow range of the nucleotide-protein interactions and none of these studies concerns prediction of nucleotide-binding sites. Although there are no methods that specifically focus on the prediction of the nucleotide-binding sites, over a dozen methods were proposed for the structure-based prediction of binding sites for small organic compounds. These methods were systematically surveyed in [3]. These methods include the geometry-based SURFNET [4], PocketFinder [5], PASS [6], LIGSITE^csc^ [7], PocketPicker [8], ConCavity [9], Fpocket [10], the energy-based Q-SiteFinder [11], the threading-based Findsite (Skolnick and Brylinski, 2008), and the consensus-based MetaPocket [12]. In this study, we aim at developing a predictor that takes protein structure as input and outputs a number of predicted nucleotide-binding sites. The proposed predictor, referred to as NSiteMatch, integrates the geometrical analysis, energy calculation, and template comparison into a single algorithm. The interaction between proteins and nucleotides only involves a few residues of the binding site. Therefore, it is likely that proteins folding into distinct overall structures employ similar binding site. NSiteMatch aims at identifying similarity between local surface patches; that is, it calculates a similarity score between a binding site (template) and a surface patch of the target protein. The similarity score considers both the arrangement of the binding residues and the shape of the pocket, in which the ligands are located. Subsequently, an energy function is employed to assess the interaction between a surface patch and the predicted ligand. Thereby, NSiteMatch concentrates the merits of geometry-, energy-, and threading-based algorithms and potentially achieves higher success rates than pervious methods.

## 2. Methods

The input of NSiteMatch method is a protein structure with coordinates of all nonhydrogen atoms while the outputs are the coordinates which represent the centers of the predicted binding sites. For instance, if NSiteMatch identifies three binding sites for a given protein structure, the output would be the centers of the three binding pockets: (*x*_1_, *y*_1_, *z*_1_), (*x*_2_, *y*_2_, *z*_2_), and (*x*_3_, *y*_3_, *z*_3_). The dataset preparation, the NSiteMatch algorithm, and the evaluation protocols are given as follows.

### 2.1. Dataset Preparation

The benchmark dataset is designed to cover a wide range of nucleotide-binding proteins. The nucleotides that are considered in this study contain at least one of the five nucleobases, a 5-carbon sugar, and 1 to 3 phosphates. We extracted all complexes from PDB that include these nucleotides [13]. Next, the maximal pairwise sequence identity of the resulting protein chains for each of the nucleotides was reduced to 40% with CD-hit (Li and Godzik, 2006). We include the nucleotides that bind to at least 50 chains where these chains belong to at least 20 different superfamilies based on the SCOP classification [14]. The availability of at least 50 chains provides us with a sufficient number of annotated binding sites to build and evaluate a well-performing predictor. While the availability of at least 20 superfamilies assures that these nucleotides bind to a wide range of proteins that are diverse in their structure and sequence, the latter is based on the 40% sequence similarity filtration. This also allows us to investigate the prediction of the distant functional relationships, that is, binding of the same nucleotides to structurally different proteins. We extracted a total of 227, 321, and 140 chains that bind to ATP, ADP, and AMP, respectively. The other nucleotide types were excluded due to the small sample size. Since the NSiteMatch and Findsite predictors utilize a template library which could contain structures that are (too) similar to the predicted protein, we created a reduced version of the dataset that contains protein chains annotated using the SCOP labels for each of the three nucleotides. In other words, we excluded the chains that were not included in the SCOP database. Using these annotations we could control the similarity/homology levels between the template library and the predicted protein. This allows us to assess the predictive quality of the NSiteMatch and Findsite when using templates that are dissimilar, at a given homology level, to the predicted protein. As a result, we extracted a total of 114, 158, and 66 chains that are annotated with SCOP labels for ATP, ADP, and AMP, respectively.

**Figure 1:**
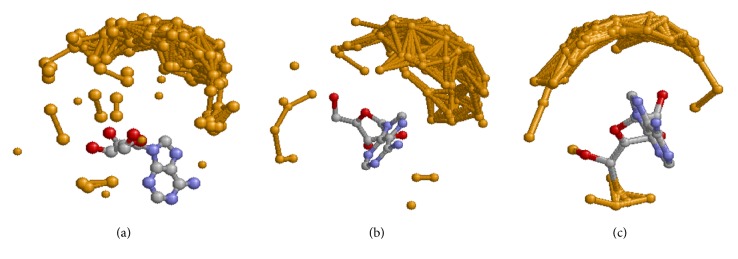
Superimposing of the complexed ATP (a), ADP (b), and AMP (c) structures. In (a), the Adenine structures of all ATPs are superimposed while the phosphorus atoms of the *γ*-phosphate (colored orange) are scattered in the space. In (b), the Adenine structures of all ADPs are superimposed while the phosphorus atoms of the *β*-phosphate (colored orange) are clustered in a small range of the space. In (c), the Adenine structures of all AMPs are superimposed while the phosphorus atoms k2of the *α*-phosphate (colored orange) are clustered in a small range of the space. It should be noted that the bond between phosphorus atoms is introduced by molecular visualization software and is not a real bond.

We have analyzed the orientation of the complexed ligands; see Figure 1. For ATP (Figure 1(a)), the Adenine structures are superimposed while the phosphorus atoms of the *γ*-phosphate (colored orange) are displayed to represent the orientation of the ligand. Figure 1 shows that the phosphorus atoms of the *γ*-phosphate are scattered in the space. For ADP, the Adenine structures are also superimposed; see Figure 1(b). The phosphorus atoms of the *β*-phosphate (colored orange) are clustered in a small range of the space. Similarly, we have superimposed the Adenine structures of all AMPs (Figure 1(c)). The phosphorus atoms of the *α*-phosphate (colored orange) are clustered in a small range of the space. Clearly, the geometries of all three ligands are confined to a certain range. We have also performed a simple statistics on the number of contacts between different functional groups of ligand and binding site. For ATP, the  *γ*-, *β*-, and *α*-phosphates and the Adenine on average form 6, 6, 4.8, and 14.3 contacts with binding sites, respectively. For ADP, the *β*- and *α*-phosphates and the Adenine on average form 5.7, 4.9, and 15.1 contacts with binding sites, respectively. For AMP, the *α*-phosphate and the Adenine on average form 5.4 and 15.3 contacts with binding sites. Clearly, for ATP-binding, both the phosphates and Adenine play an important role. However, for ADP- and AMP-binding, the Adenine is more involved in the interaction when compared with the phosphates.

### 2.2. Preparation of the Template Library of NSiteMatch

The template library of NSiteMatch consists of the structures of the nucleotide-binding sites. For a given protein-nucleotide complex, a nonhydrogen atom of the protein is considered as an interacting atom if it is within 3.9 Å of a nonhydrogen atom of the nucleotide [15]. A binding site is defined as a collection of the interacting atoms that bind to the same nucleotide molecule. The 3D-coordinates, the atom types, and the residue types of the interacting atoms of each binding site were stored in the template library.

### 2.3. The NSiteMatch Algorithm

The novelty of our approach is twofold. First, the NSiteMatch combines the geometrical, energy-based, and threading approaches. Second, drawing from the observation that use of templates with the sufficient structure similarity leads to high quality predictions, we use a template database to perform the predictions. However, unlike the only existing threading-based Findsite that relies on the overall similarity of the entire protein fold, we use local similarity of the structure in the binding region to find the most suitable templates. This allows us to identify a larger number of potentially useful templates and to predict distant functional relationships, that is, NSiteMatch utilizes the templates that share similarity in the binding region but which may share low homology with the predicted protein.

The NSiteMatch method includes two major phases. The first phase (Steps 1–8) performs fitting of templates into the structure of the predicted protein based on a common substructure defined by the interacting atoms (Steps 1–8); this is repeated for each template and each potential position of the center of the ligand. The second phase (Steps 9 and 10) processes the predictions, which are filtered using a docking energy function based on AMBER force field, and next they are clustered and ranked. The method outputs the ranked list of the predicted centers of the ligands and the corresponding ranked list of the binding residues for each center. The above overview demonstrates that our method combines the geometrical approach, which is implemented in the procedures to define and score the templates, energy-based approach, by utilizing energy function to filter initial predictions, and threading, by using the template database.

**Figure 2:**
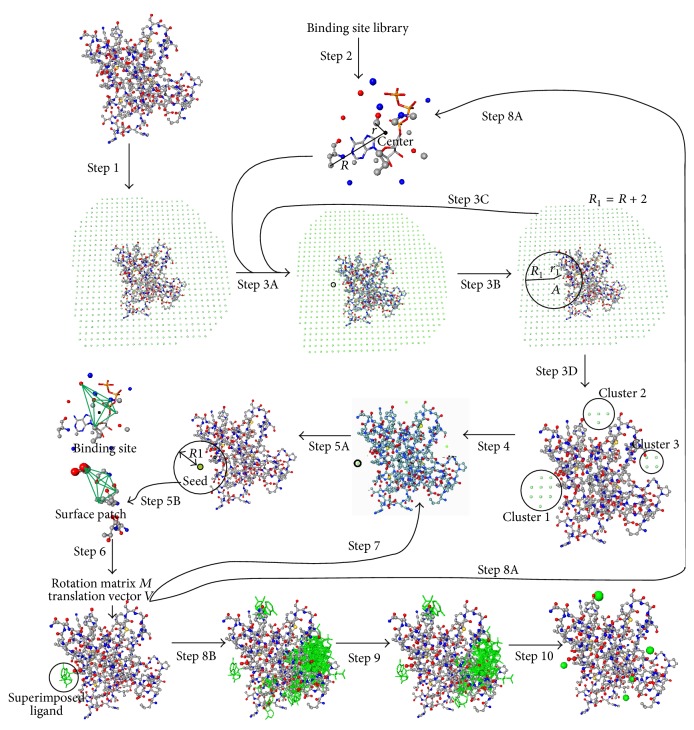
The overall flow of the NSiteMatch algorithm, which includes 10 steps. The details of the algorithm are given in Methods.

The overall flow of the NSiteMatch algorithm is given in Figure 2. Given the predicted protein structure and a template library with the nucleotide-binding sites, the NSiteMatch algorithm is implemented with the following 10 steps.

**Step 1 (set the 3-dimensional grid space for the predicted protein).**

We use grid with a step size of 2 Å and a given grid point is retained if it is within 10 Å to a nonhydrogen atom of the protein. A grid point is marked as protein and removed from the grid space if it is within 1.6 Å to a nonhydrogen atom of the protein; otherwise, the grid point is kept and annotated as solvent.

**Step 2 (select a binding site from the template library).**

The binding site contains both the coordinates of the interacting atoms of the protein and the coordinates of the nucleotide atoms. We calculate the geometrical center of the nucleotide and the distances between the center and all interacting atoms of the protein. We use these distances to set values of two parameters. Among these distances, the maximal distance *R* represents the radius to cover all interacting atoms while the minimal distance *r* represents the distance between the center and the protein surface. The two parameters *R* and *r* are used in the subsequent steps.

**Step 3 (scan the grid space to assess which grid points fit the geometrical center of the binding site).**

In step  3A, we first choose a grid point from the grid space. Next, we assess whether the chosen grid point fits the geometrical center of the binding site, which is performed in step  3B. In step  3B, we first calculate the distances between this grid point and all atoms of the protein. Among these distances, the minimal distance is denoted as *r*_1_. Our first premise is that if a grid point fits the geometrical center of a nucleotide, the distance between the grid point and the protein surface should be similar to the distance *r* between the center of the nucleotide and the protein surface. Therefore, a given grid point is retained only if |*r* − *r*_1_| ≤ 2 Å. The 2 Å margin is used to accommodate the step size of the grid space. Our second premise is that if a grid point fits the center of a nucleotide, the spatial arrangements of interacting atoms (atoms that participate in the protein-ligand interaction) around this point should be similar to the arrangements of atoms around the center of the nucleotide. We use triangles, of which two vertexes are the interacting atoms of the protein and the third vertex is the grid point (the third vertex is the center of the nucleotide in the template), to represent this spatial arrangements. For a given grid point, the grid-associated surface patch is defined as a collection of protein atoms that are within *R*_1_ = *R* + 2 Å to the grid point. By this definition, the radius *R*_1_ of the grid-associated surface patch is slightly larger but still similar to the radius *R* of the binding site. We compare the triangles formed by the atoms of the template binding site and the triangles formed by the atoms of the grid-associated surface patches. The triangles are formed by two atoms of the grid-associated surface patch (or template binding site) and the grid point (or the center of the nucleotide). Among the vertexes, the grid point or the center of the nucleotide is invariant while the other two vertexes are chosen from a large number of combinations of the corresponding atoms. Therefore, we generate two sets of triangles for the binding site and the grid-associated surface patch. We say that a given triangle of the grid-associated surface patch matches a given triangle of the template binding site if the corresponding vertexes have the same atom type and residue type and the difference between the side length of the corresponding edges is less than or equal to 2 Å. A grid point and the associated surface patch are retained if at least 25% of the triangles in the template site match with the triangles of the surface patch, and the surface patch matches at least 50 triangles of the template binding site. In step  3C, we go back to step  3A and choose another grid point until all grid points are used. Finally, in step  3D, the retained grid points are passed to Step 4.

**Step 4 (cluster the retained grid points).**

Two retained grid points are assigned to the same cluster if they are neighboring grid points, that is, the distance between the two points is 2 Å. The clusters are sorted by the number of grid points and the top three clusters are selected; if the total number of clusters is smaller than 3, then all clusters are selected. We use two points to represent each cluster: the geometrical center of the grid points and the point with the maximal number of triangles that match the template binding site. We refer to these representative grid points as seeds and the associated surface patches as seed-associated surface patches.

**Step 5 (search for the maximal common substructure between the binding site and the seed-associated surface patches).**

The seed-associated surface patch is defined as the collection of protein atoms that are within *R*_1_ = *R* + 2 Å to the seed (grid point); see step  5A. We search for the maximal common substructure between the template binding site and the seed-associated surface patches. We denote the atoms at the template binding site as *a*_1_, *a*_2_,…, *a*_*n*_ and the atoms at the seed-associated surface patch as *b*_1_, *b*_2_,…, *b*_*m*_. An atom from the template site matches an atom from the surface patch if the two atoms have the same atom type and residue type. For every pair of the matched atoms, we create a corresponding vertex *g*(*a*_*i*_, *b*_*j*_) on a new graph *G*, where *a*_*i*_ is the atom from the binding site and *b*_*j*_ is the atom from the selected surface patch and *a*_*i*_ matches *b*_*j*_. Two vertices *g*_*k*_(*a*_*i*_, *b*_*j*_) and *g*_*l*_(*a*_*s*_, *b*_*t*_) in graph *G* are connected if two conditions are satisfied. First, |*D*(*a*_*i*_, *a*_*s*_) − *D*(*b*_*j*_, *b*_*t*_)| ≤ 2 Å, where *D*(*a*_*i*_, *a*_*s*_) is the distance between *a*_*i*_ and *a*_*s*_ and *D*(*b*_*j*_, *b*_*t*_) is distance between *b*_*j*_ and *b*_*t*_. Second, *a*_*i*_ ≠ *a*_*s*_ and *b*_*j*_ ≠ *b*_*t*_. Searching for the maximal common substructure between the template binding site and a given surface patch is equivalent to searching for the complete subgraph in *G*. We used the backtracking algorithm to search for the complete subgraph [16]. The identified common substructure (atoms connected with green solid lines) between the template binding site and the seed-associated surface is shown in step  5B.

**Step 6 (superimpose the template binding site into the seed-associated surface patches).**

In Step 5 we identified a common substructure between the template binding site and a given seed-associated surface patch. By using the coordinates of the two substructures, we calculated the RMSD value between the two substructures and the translation vector (*V*) and the rotation matrix (*M*) to achieve this RMSD value. Based on *V* and *M*, we superimpose the nucleotide structure at the template binding site into the corresponding surface patch.

**Step 7 .**

*Select the next available seed and repeat Steps* 5 *and* 6 *until all seeds are used. *

**Step 8 .**

*Select the next available binding site in the template library and repeat Steps* 2 *–* 6 *until all templates in the library are used*; see step  8A. Once all templates are used, step  8B passes the predictions (the locations of the binding nucleotides) to Step 9.

**Step 9 (filter the predictions by using a docking energy function).**

Our algorithm superimposes a number of nucleotide structures on the surface of the predicted protein. The putative coordinates of the nucleotides are calculated by using the translation vector and the rotation matrix obtained in Step 6. The conformation of the superimposed nucleotide is copied from the original binding site. Hence, both the coordinates of the protein and the putative coordinates of the superimposed nucleotides are known. Therefore, we can assess the predictions based on docking energy functions. We use the AMBER force field for energy calculation [17]. Since protein and the nucleotides are not covalently linked, we only considered van der Waals and electrostatic energies. The predicted nucleotide structures with an energy that suggests weak interaction between this nucleotide and the protein are discarded.

**Step 10 (generate the predicted binding sites and binding residues).**

The NSiteMatch method predicts both binding sites and binding residues. For each of the superimposed nucleotide structures, we calculate its geometrical center and the residues that interact with this structure. The binding sites and binding residues are generated separately. For the generation of the binding sites, the geometrical centers are clustered based on the distances between them. Two geometrical centers are assigned to the same cluster if the distance between them is less than 4 Å. The clusters are ranked by the number of centers of each cluster. We use the geometrical center of all centers of one cluster to represent this cluster. The geometrical centers of the top *n* clusters are outputted as the predicted binding sites; by default *n* = 5. For each residue in the predicted protein, we count the number of nucleotide structures that the residue interacts with. The residues are sorted and scored by these counts in the descending order. The scores are used to annotate a given residue as binding or nonbinding based on a cutoff threshold. We selected 2 thresholds that result in predictions that match the highest precision or recall, respectively, achieved by the other methods, including Findsite, MetaPocket, and Q-SiteFinder.

### 2.4. Evaluation Measures and Setup

The NSiteMatch, Findsite, MetaPocket, and Q-SiteFinder generate both the coordinates of the binding sites and the list of the binding residues. Therefore, their predictions are evaluated at two levels as follows.

#### 2.4.1. Evaluation of the Predicted Coordinates of the Binding Sites

It is done by using *D*_*CC*_, which is the minimal* distance* from the* center* of the predicted binding site to the* center* of the ligand. The *D*_*CC*_ index was used in the evaluation of binding site predictors in a few recent studies (Skolnick and Brylinski, 2008). For a given predicted protein with *n* native nucleotide-binding sites we take the top *n* predictions for each of the four considered methods. A given binding site is assumed to be correctly predicted if the minimal *D*_*CC*_ between this site and any of the *n* predictions from a given method is below a threshold *D*. We calculate a success rate over the entire dataset for a given value of *D*, which is defined as the number of correctly predicted binding sites divided by the total number of sites.

#### 2.4.2. Evaluation on the Predicted Binding Residues

A given residue is defined as the binding residue if a nonhydrogen atom of that residue is within 3.9 Å to a nonhydrogen atom of the nucleotide. The same 3.9 Å threshold was used in the investigation of protein-DNA and protein-small ligand interactions [15]. For a given predicted protein we extract the binding residues for the top *n* predictions generated by each of the four methods and we compare them with the native binding residues using the following three measures:

(1) Precision PREC=TPTP+FPRecall REC=TPTP+FNMCC=TP∗TN–FP∗FNsqrtTP+FP∗TP+FN∗TN+FP∗TN+FN,

where TP (true positives) and TN (true negatives) are the counts of correctly predicted binding and nonbinding residues, respectively, FP (false positives) are the nonbinding residues that were predicted as the binding residues, and FN (false negatives) are the binding residues that were predicted as the nonbinding residues.

The NSiteMatch method is compared with the Findsite, MetaPocket, and Q-SiteFinder on three benchmark datasets that concern ADP-protein, ATP-protein, and AMP-protein interactions, respectively. The NSiteMatch and Findsite are template-based methods and therefore the predictive quality of these two methods depends on the similarity between the predicted protein and the template library. We use four filters to assess the ability of these two methods to predict binding sites on proteins that are dissimilar to the template library. For a predicted protein, we use only the template structures that share at most 40% sequence similarity and that are in a different protein family, superfamily, and fold to the predicted protein, respectively; the latter three filters are based on the SCOP annotations [14]. Proteins that lack the SCOP labels were not used to perform the evaluation for the homology-based filters, but they were used to assess with the 40% identity filter. We note that the first, sequence similarity-based filter may use templates from the same family. A study that analyzed SCOP annotations demonstrated that pairs of proteins that share > 25% sequence similarity are assigned to the same protein family in 99% of the cases [18]. Similarly, the CATH database [19], which is another protein homology classification system, automatically assigns two proteins that share > 35% sequence similarity to the same family. The evaluation of the NSiteMatch and Findsite is based on the jackknife test, where each protein in the dataset is selected once as the test/predicted protein and the remaining chains are used as the template library.

The predictions for the template-free MetaPocket and Q-SiteFinder were performed using the corresponding web servers. This means that it is possible that some of the predicted proteins were used to build these predictive models. This should not lead to a significant advantage since both of these methods use prediction models that do not utilize templates and which were computed using a large dataset of diverse proteins-ligand complexes.

### 2.5. Statistical Analysis

The statistical analysis follows the procedures for the comparative analysis of existing binding site predictors. For a protein with *n* binding sites we take the top *n* predictions for every considered prediction method. For each of the *n* binding sites, the minimum distance is calculated between this site and the top *n* predictions. Consequently, for a dataset with *m* binding sites, a set of minimal distances {*d*_*i*_; *i* = 1,2,…, *m*} are generated for each method. We assume that the predictions from different methods that are farther than 10 Å away from the native site are equally wrong, that is, they are too far away to be meaningful, and thus we round them down to 10 Å. The significance of the differences between a given pair of predictors is measured by evaluating the corresponding, for the same *m*, minimal distance values. Since the distances for the considered predictors are not normally distributed, we use the nonparametric Wilcoxon signed-rank test. We assume that the differences are significant if *p* < 0.05.

## 3. Results and Discussion

### 3.1. Evaluation of the Predicted Binding Sites

**Figure 3:**
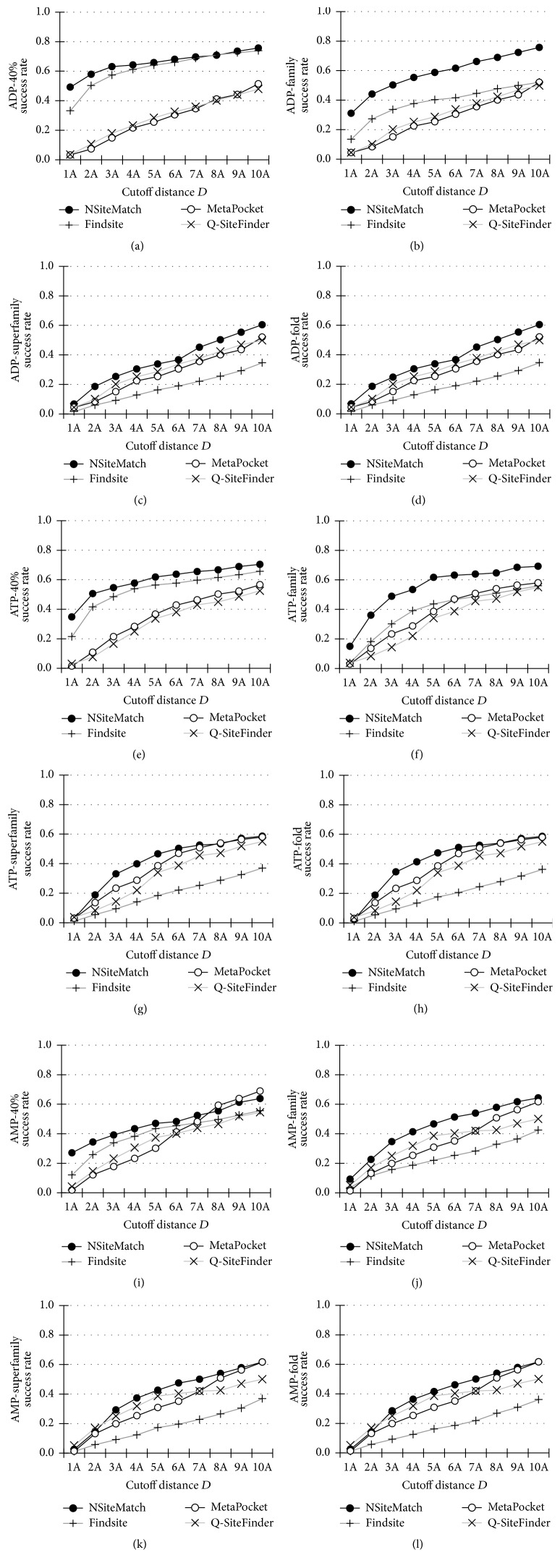
The success rates (*y*-axis) of the NSiteMatch and the three competing methods (Findsite, MetaPocket, and Q-SiteFinder) measured using *D*_*CC*_ (the minimal distance from the center of the predicted site to the center of the ligand) on the benchmark datasets. A given binding site is regarded as correctly predicted if the minimal distance between this site and the top *n* predictions is below the cutoff distance* D* (*x*-axis), where *n* is the number of binding sites of the protein that includes the evaluated binding site. All methods are evaluated at 4 filter levels, the 40% sequence similarity level ((a), (e), and (i)), family level ((b), (f), and (j)), superfamily level ((c), (g), and (k)), and fold level ((d), (h), and (l)). (a), (b), (c), and (d) show results for the ADP. (e), (f), (g), and (h) show results for the ATP and (i), (j), (k), and (l) show results for the AMP. The 40% sequence similarity level indicates that all chains in the template library that were used for the prediction share less than 40% sequence similarity to the test protein. The family, superfamily, and fold levels indicate that all chains in the template library that were used for the prediction are classified as belonging to a different family, superfamily, and fold (annotated using the SCOP database), respectively, when compared with the annotation of the test protein.

The success rates of NSiteMatch, Findsite, MetaPocket, and Q-SiteFinder quantified using *D*_*CC*_, which measures the distance from the center of the predicted site to the center of the ligand in its native location, are shown in Figure 3. For each nucleotide type, the success rates are calculated using the four filters: the 40% sequence similarity and the family-, superfamily-, and fold-based homology.

For the 40% sequence similarity filter, the NSiteMatch and Findsite achieve higher success rates than the Q-SiteFinder and MetaPocket for the three types of the nucleotides; see Figures 3(a), 3(e), and 3(i). For the cutoff *D* = 4 Å, which was suggested by Skolnick and colleagues (Skolnick and Brylinski, 2008), the success rates of NSiteMatch, Findsite, MetaPocket, and Q-SiteFinder are 64%, 61%, 22%, and 23% for the ADP; 58%, 54%, 28%, and 25% for the ATP; and 43%, 38%, 23%, and 31% for the AMP, respectively. When considering the cutoff distances *D* between 1 Å and 5 Å, NSiteMatch achieves 7-8%, 16–43%, and 21–45% higher success rates than the Findsite, Q-SiteFinder, and MetaPocket, respectively.

At the family level, the NSiteMatch again outperforms the remaining methods; see Figures 3(b), 3(f), and 3(j). For the cutoff *D* = 4 Å, the success rates of NSiteMatch are 55%, 53%, and 41% for the ADP, ATP, and AMP, respectively. To compare, the corresponding success rates for the Findsite, Q-SiteFinder, and MetaPocket are 38%, 25%, and 23% for the ADP; 39%, 22%, and 29% for the ATP; and 19%, 32%, and 25% for the AMP. Although Findsite obtains higher success rates than the Q-SiteFinder and MetaPocket for the ADP and ATP, its success rates for the AMP are lower than the rates of the other two methods. This is likely because the template library for the AMP is smaller than the libraries for the ADP and ATP. When we exclude the proteins for which the SCOP label is not assigned and thus which cannot be used for the prediction when the homology filter is applied, the template libraries contain 158, 114, and 66 structures for the ADP, ATP, and AMP, respectively. The number of the available templates is even smaller once we also exclude the structures that belong to the same family as the predicted protein. Consequently, the lower success rates of Findsite and NSiteMatch for the AMP, when compared with the ATP and ADP, are due to the fact that fewer templates can be used. Moreover, the rates of the Q-SiteFinder and MetaPocket are relatively similar across the three nucleotides since these methods do not utilize templates. The Q-SiteFinder and MetaPocket methods also should not be sensitive to the filter.

The results at the superfamily and fold levels are similar; see Figures 3(c), 3(d), 3(g), 3(h), 3(k), and 3(l). Although the NSiteMatch still achieves higher success rates than the remaining methods, the corresponding improvements are smaller. When considering the cutoff distances *D* between 1 Å and 5 Å, the NSiteMatch achieves 14–20%, 1–12%, and 7-8% better success rates than the Findsite, Q-SiteFinder, and MetaPocket, respectively. We observe that, at the superfamily and fold filter levels, the success rates of the Q-SiteFinder and MetaPocket are higher than the success rates of Findsite.

**Figure 4:**
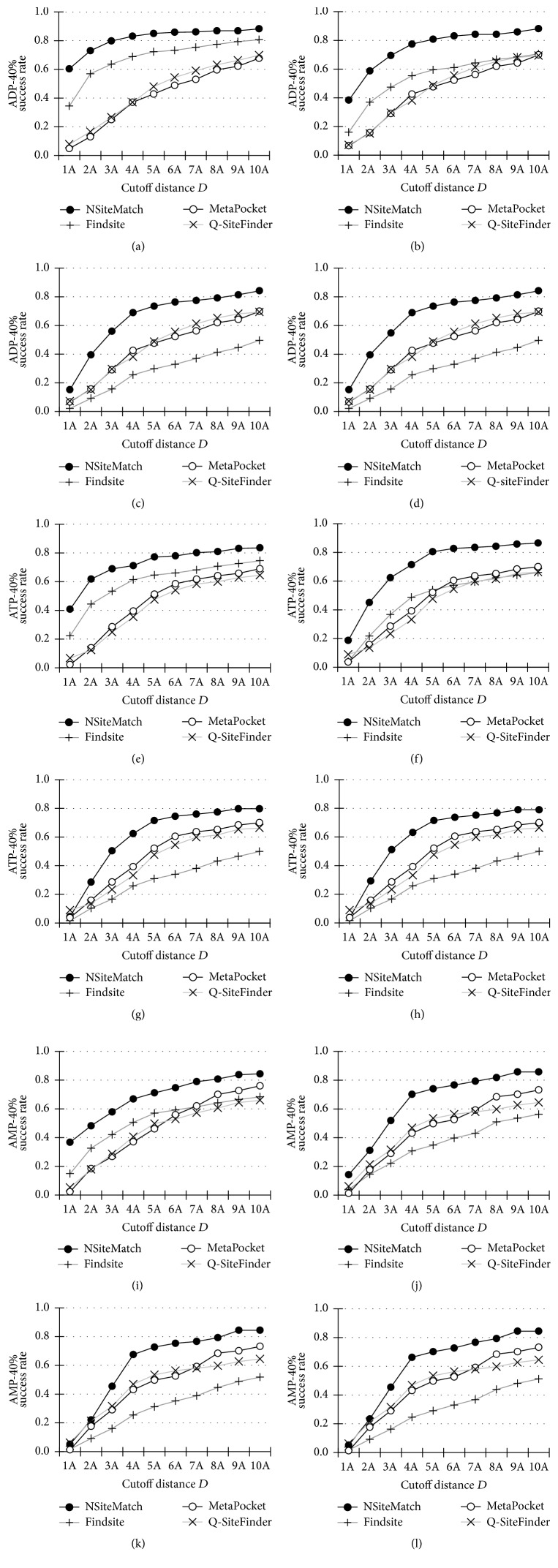
The success rates (*y*-axis) of the NSiteMatch and the three competing methods (Findsite, MetaPocket, and Q-SiteFinder) measured using *D*_*CC*_ (the minimal distance from the center of the predicted site to the center of the ligand) on the benchmark datasets. A given binding site is regarded as correctly predicted if the minimal distance between this site and the top 5 predictions is below the cutoff distance* D* (*x*-axis). All methods are evaluated at 4 filter levels, the 40% sequence similarity level ((a), (e), and (i)), family level ((b), (f), and (j)), superfamily level ((c), (g), and (k)), and fold level ((d), (h), and (l)). (a), (b), (c), and (d) show results for the ADP. (e), (f), (g), and (h) show results for the ATP and (i), (j), (k), and (l) show results for the AMP. The 40% sequence similarity level indicates that all chains in the template library that were used for the prediction share less than 40% sequence similarity to the test protein. The family, superfamily, and fold levels indicate that all chains in the template library that were used for the prediction are classified as belonging to a different family, superfamily, and fold (based on the SCOP database), respectively, when compared with the annotation of the test protein.

We also calculate the success rates of the NSiteMatch, Findsite, MetaPocket, and Q-SiteFinder quantified using *D*_*CC*_ by taking the top 5 predictions for every predicted protein; see Figure 4. Although the success rates of the four methods are improved due to the inclusion of additional predictions, the relative ranking does not change when compared with the evaluations based on the *n* predictions. For instance, at the 40% sequence similarity level, the NSiteMatch achieves success rates that are higher than the rates of the other three methods, and Findsite is the runner-up. At the superfamily and fold filter levels, the NSiteMatch outperforms the MetaPocket and Q-SiteFinder, which in turn outperforms Findsite.

**Figure 5:**
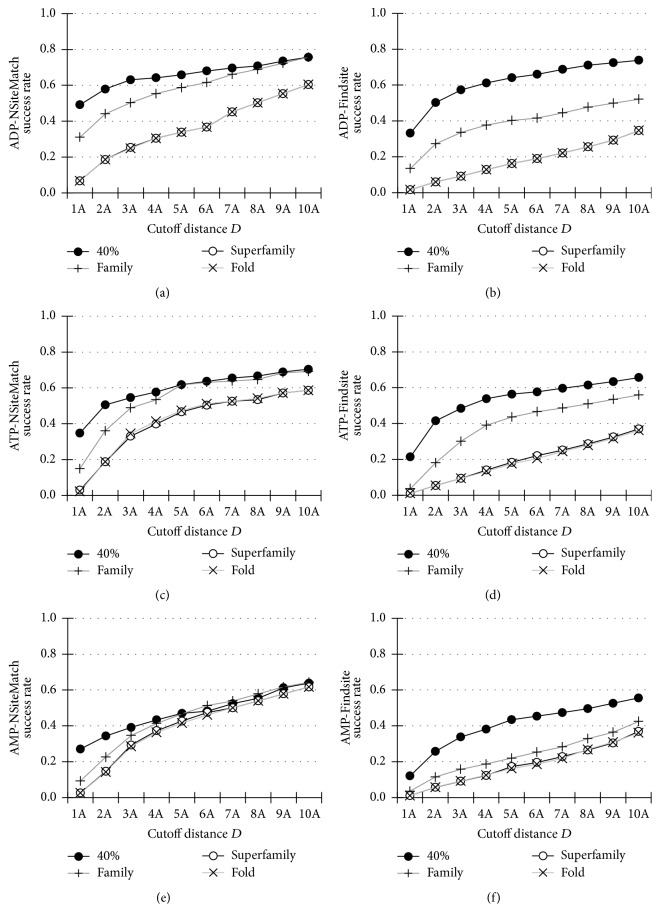
The relation between the predictive quality of the NSiteMatch and Findsite and the similarity between the predicted protein and template library. The success rates (*y*-axis) are measured using *D*_*CC*_ (the minimal distance from the center of the predicted site to the center of the ligand) on the benchmark datasets. A given binding site is regarded as correctly predicted if the minimal distance between this site and the top *n* predictions is below the cutoff distance* D* (*x*-axis), where *n* is the number of binding sites of the protein that includes the evaluated binding site. (a), (c), and (e) evaluate results of the NSiteMatch for the ADP, ATP, and AMP, respectively; (b), (d), and (f) summarize the corresponding results for the Findsite.

Among the four predictors, the NSiteMatch and Findsite are template-based and therefore they depend on the availability of suitable templates. As expected, Figure 5 reveals that the predictive quality of these two methods declines with the decrease of the structure similarity to the templates, that is, when more distant homologs are used. For instance, when considering the cutoff *D* = 4 Å, the success rates of the NSiteMatch are 64%, 55%, 31%, and 31% for the ADP at the 40% sequence similarity, family, superfamily, and fold filter levels, respectively (Figure 5(a)). Similarly, for the ATP and AMP, the success rates of the NSiteMatch are 58% and 43%, 53% and 41%, 40% and 37%, and 41% and 36% for the four filters, respectively (Figures 5(b) and 5(c)). Similar declining trends are observed for the Findsite; see Figures 5(d), 5(e), and 5(f). Although the results indicate that the availability of similar templates has a relatively strong impact on the predictive quality of these two predictors, we note that the NSiteMatch maintains higher success rates when making predictions with the help of more distant homolog. This advantage is due to the use of local similarity and consequently, as shown in Figure 3, our method also outperforms the two template-free methods, MetaPocket and Q-SiteFinder, at the superfamily and fold levels even for the hard (characterized by the small template library) AMP ligand. To compare, these two approaches outperform Findsite when the templates are filtered at the superfamily and fold levels for each of the three ligands.

We investigate significance of differences in the prediction quality measured with *D*_*CC*_ between NSiteMatch and the other predictors; see Table 1. We compare the *D*_*CC*_ values that are calculated by taking top *n* predictions for each protein where *n* is the number of the nucleotide-binding sites for a given protein. At 40% sequence similarity and family levels, the NSiteMatch is significantly better than the other three methods. Similarly, our method significantly outperforms the competing solutions by using the superfamily and fold filters for the ADP and ATP ligands, and the improvements are not significant only when compared with the MetaPocket and Q-SiteFinder for the AMP.

### 3.2. Evaluation of the Predicted Binding Residues

Besides the coordinates of the predicted binding site, the NSiteMatch, Findsite, MetaPocket, and Q-SiteFinder also predict the binding residues. For the NSiteMatch, each residue in the predicted protein structure is assigned with a numerical score which indicates the number of ligands that this residue interacts with (details concerning the annotation of the binding residues for the NSiteMatch are given in Methods). A given residue is regarded as a binding residue if its score is above a certain threshold. The selection of this threshold controls the trade-off between precision (fraction of the correctly predicted binding residues among all predicted binding residues) and recall (fraction of the correctly predicted binding residues among all native binding residues). Since the precision and recall values achieved by the Findsite, MetaPocket, and Q-SiteFinder vary substantially, we selected two thresholds that allow for a direct comparison. Similarly as in Zhang et al., 2008, we set the threshold such that the precision/recall of the NSiteMatch is equal to the highest precision/recall achieved by the other methods for a given ligand and a given filter. The predictions are evaluated based on the recall (also called sensitivity), precision, and MCC; see Table 2. The MCC quantifies correlation between predictions and the native annotations and thus higher MCC values correspond to more accurate predictions.

For the 40% sequence similarity filter, the NSiteMatch achieves higher precision, recall, and MCC values than the Findsite, Q-SiteFinder, and MetaPocket for all three types of the nucleotides. The NSiteMatch generates predictions with a substantially higher precision when its recall is the same as the highest recall produced by the other predictors. Similarly, our method has higher recall when its precision matches the highest precision produced by the other methods. Findsite obtains the second best MCC values for the three types of the nucleotides. We observe that predictions of MetaPocket are characterized by the precision that is higher than the recall, while Q-SiteFinder has the recall values higher than the precision. This indicates that MetaPocket and Q-SiteFinder under- and overpredict the binding residues, respectively.

At the family level, NSiteMatch also provides the highest precision, recall, and MCC values when compared with the other methods for the three nucleotides. However, as expected, the predictive quality of NSiteMatch and Findsite declines when compared to the 40% sequence similarity filter. Based on the MCC value, Findsite outperforms the Q-SiteFinder and MetaPocket for the ADP and ATP but is inferior to Q-SiteFinder for the AMP. The results at the superfamily and fold level filters are similar to each other. The NSiteMatch maintains the highest precision, recall, and MCC values for the ADP and ATP. However, for AMP, the predictions of the NSiteMatch have quality that is comparable to Q-SiteFinder and higher than the MetaPocket and Findsite.

The results concerning prediction of binding residues are consistent with our statistical analysis based on the *D*_*CC*_ values. We note that NSiteMatch and Findsite achieve lower predictive quality for AMP, when compared with ADP and ATP. This is likely because ADP and ATP contain more phosphates than AMP, while the phosphates form strong interaction with binding site through long range electrostatic interactions. In the case of AMP, the Adenine nucleoside plays a more important role for interacting with binding site. However, the hydrogen bonding from the hydroxyls of the ribose ring and from the Adenine heterocycle is much weaker when compared with the electrostatic interaction. The results suggest that prediction of the binding site of Adenine is more difficult than prediction of binding sites of phosphates.

Similarly as for the prediction of the binding sites, we assessed the impact of the similarity between the predicted protein and the corresponding template library on the predictive qualities of the NSiteMatch and Findsite for the prediction of binding residues. The MCC values achieved by NSiteMatch for the ADP, ATP, and AMP are 0.6, 0.56, and 0.51, respectively, at the 40% sequence similarity level; 0.52, 0.47, and 0.39, respectively, at the family level; 0.44, 0.41, and 0.35, respectively, at the superfamily level; and 0.43, 0.41, and 0.34, respectively, at the fold level. As expected, the results indicate that NSiteMatch generates better predictions when the predicted protein has a higher structural similarity to the template library. A similar relation is observed for the Findsite; see Table 2. However, based on the MCC values, the Findsite is outperformed by the template-free Q-SiteFinder for the prediction of binding residues of the three nucleotides for the superfamily and fold filters. Importantly, we observe that our method outperforms Findsite for each filter and each ligand type, and it also improves over the Q-SiteFinder and MetaPocket, except for the AMP with the superfamily and fold level filters where it provides predictive quality that is comparable to the Q-SiteFinder. This demonstrates that our local similarity-based approach provides one of the best solutions for the structure-based prediction of nucleotide-binding residues, even when predicting for structures from novel/uncharacterized folds and superfamilies.

### 3.3. Case Studies

We present two case studies. The first compares the utility of the NSiteMatch and the existing binding site predictors, and the second demonstrates the ability of the NSiteMatch to identify similar binding sites across protein folds.

**Figure 6:**
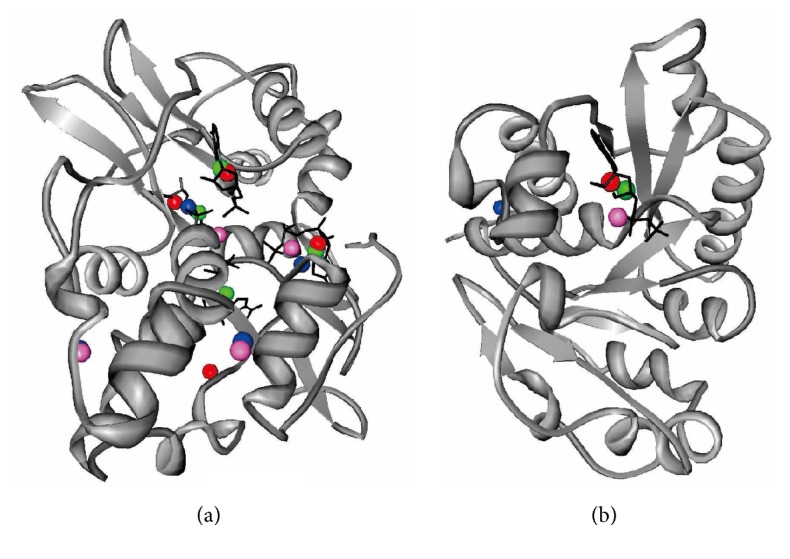
Binding sites predicted by the NSiteMatch, Findsite, MetaPocket, and Q-SiteFinder for chain A of the MJ1225 protein (a) and chain A of the cell division inhibitor mind protein (b). The predictions by NSiteMatch, Findsite, MetaPocket, and Q-SiteFinder are denoted with green, red, purple, and blue spheres, respectively. The ligands are in the stick format and are colored in black. The MJ1225 contains 3 ADP-binding sites and 1 AMP-binding site and the top 4 predictions from each method are shown. The cell division inhibitor mind protein has 1 ADP-binding site and the top prediction for each method is shown.

We use the chain A of the MJ1225 protein (PDB code: 3KH5) [20] for the first case study. This structure was released after our benchmark dataset was created and this sequence shares less than 25% similarity to any sequence in our benchmark dataset. We used the web servers of the MetaPocket and Q-SiteFinder and the standalone implementation of the Findsite and our NSiteMatch to generate the predictions. The template library of Findsite and NSiteMatch includes all structures from the benchmark dataset. Since the MJ1225 protein includes 3 ADP-binding sites and 1 AMP-binding site, the top 4 predictions generated by each predictor were assessed. For the cutoff distance *D* = 4 Å, the NSiteMatch and Findsite correctly predict 4 and 3 of the binding sites, respectively, while the Q-SiteFinder and MetaPocket find 2 and 1 of the binding sites, respectively; see Figure 6(a). The lower quality of the Q-SiteFinder and MetaPocket predictions can be explained by the fact that these methods predict sites for a generic class of small ligands, while Findsite and NSiteMatch use a library that is specific to the three nucleotides. In spite of using the same template library, we show that NSiteMatch is more accurate than Findsite, which is due to the use of the local similarity.

**Figure 7:**
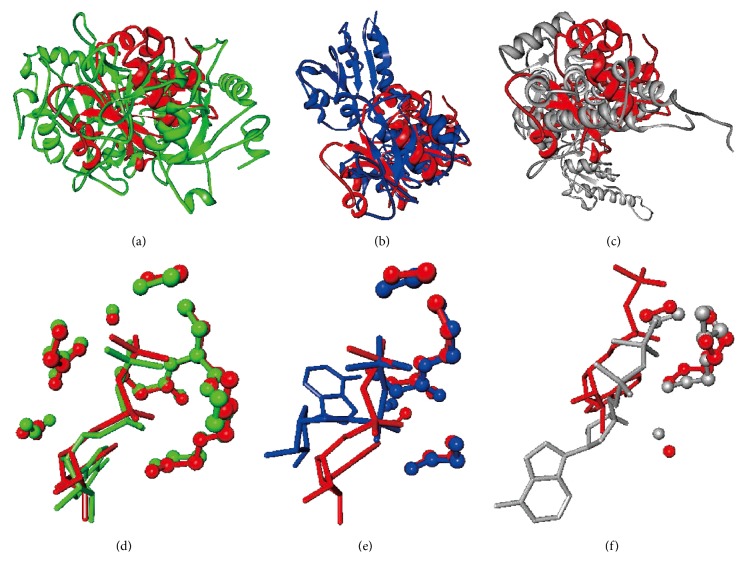
Comparison of the structures of templates identified by the NSiteMatch as similar to the chain A of the probable cell division inhibitor mind protein (PDB code: 1ION), which are classified as belonging to different superfamily as the 1ION protein. The 1ION structure is shown in red, while the three templates, phosphoenolpyruvate carboxykinase, UDP-N-Acetylmuramoylalanine-D-Glutamate ligase, and thermosome alpha subunit, are in green, blue, and grey, respectively. (a), (b), and (c) superimpose each of the templates to the 1ION structure by using Fr-TM-align. (d), (e), and (f) are the common substructures between the 1ION structure and a given template, which were identified by the NSiteMatch. The residues are displayed in the ball and stick format and the ADP is shown in the stick format.

We use chain A of the probable cell division inhibitor mind protein (PDB code: 1ION) [21] to demonstrate that NSiteMatch is capable of identifying similar binding sites across protein folds. This would imply that the function of a given protein could be inferred from other proteins that have different topologies. This structure includes 1 ADP-binding site and thus we assess the top prediction from each method. The distances between the predicted and the native center of the ligand are 0.6 Å, 1.3 Å, 3.0 Å, and 22.8 Å for the NSiteMatch, Findsite, MetaPocket, and Q-SiteFinder, respectively; see Figure 6(b). The ADP-binding site is implemented by the “GTGKTT” sequence segment and this protein is assigned to the “P-loop containing nucleoside triphosphate hydrolases” superfamily based on the SCOP annotation. The NSiteMatch uses potentially multiple templates to find a single binding site. We analyze the templates that the NSiteMatch finds as similar to the 1ION protein in the predicted binding region and which were used to predict this site. Three of these templates belong to superfamilies that are different to the superfamily of the predicted protein; see Table 3. The first template is chain A of phosphoenolpyruvate carboxykinase (PDB code: 1K3C) [22], which is assigned to the “PEP carboxykinase-like” superfamily in SCOP. The other two templates are chain A of UDP-N-Acetylmuramoylalanine-D-Glutamate ligase (PDB code: 2JFG) [23] and chain A of Thermosome alpha subunit (PDB code: 1Q3S) [24], which belong to the “MurD-like peptide ligases” and “catalytic domain and GroEL equatorial domain-like” superfamilies, respectively. We superimpose these three templates into the predicted 1ION protein using Fr-TM-align [25]; see Figures 7(a), 7(b), and 7(c). The figures reveal that the templates are dissimilar in their overall topology when compared with the 1ION protein. The alignment of the binding segments for the three templates and the predicted protein, which is given in Table 3, reveals that they share key binding residues, that is, the Gly, Lys, and Thr residues. The NSiteMatch works by finding local similarity in the binding region between the predicted and the template proteins, and we superimposed these regions; see Figures 7(d), 7(e), and 7(f) where the residues are displayed in ball and stick format and the ADP is shown in the stick format. The binding site of the phosphoenolpyruvate carboxykinase is very similar to the binding site of the predicted protein (Figure 7(d)); we found 30 atoms which overlap between these two superimposed sites. The overlap between the binding site of the UDP-N-Acetylmuramoylalanine-D-Glutamate ligase and the predicted protein includes 16 atoms (Figure 7(e)) which mainly involve the Gly114, Lys115, and Thr117 residues on the template and the Gly15, Lys16, and Thr18 residues on the predicted chain. The binding site of the thermosome alpha subunit is less similar to the predicted protein when compared with the other two templates (Figure 7(f)); 11 atoms overlap and they correspond to Gly96, Thr98, and Thr99 residues on the template and Gly15, Thr17, and Thr18 residues on the predicted sequence. We observe that the ADP binds to the predicted protein mainly through the beta-phosphate. Similarly, the ADP binds to the first two templates also mainly through the beta-phosphate, while it interacts with the third template mainly through the *α*-phosphate group, which explains the lower similarity. However, even when the interaction group changes, the NSiteMatch was still able to capture a similar spatial arrangement of residues at the binding site. This example demonstrates that our method can perform annotation of binding sites based on templates with distant homology. In contrast to the NSiteMatch, the templates used by Findsite to predict the 1ION protein belong to the same “P-loop containing nucleoside triphosphate hydrolases” superfamily; that is, Findsite was not able to capture the distant functional relationship between proteins from different superfamilies.

## 4. Conclusions

Motivated by the importance and the substantial interest in protein-nucleotide interactions and the lack of accurate computational predictors, we designed a novel and accurate structure-based nucleotide-binding site predictor for the three most commonly occurring nucleotides. However, it should be noted that the proposed NSiteMatch achieves lower predictive quality for AMP when compared with ADP and ATP. The difference between the three nucleotides is the number of phosphates. It suggests that NSiteMatch is more focused on prediction of binding sites for phosphates than Adenine and NSiteMatch should be expended to encompass the interaction pattern of the Adenine nucleoside. Empirical testing shows that the proposed NSiteMatch method significantly outperforms generic, template-free binding site predictors, except for the AMP nucleotide, for which NSiteMatch generates results that are comparable to the Q-SiteFinder and MetaPocket at the superfamily and fold filter levels. We also show that the template-based NSiteMatch and Findsite generate better predictions for proteins that share higher similarity with their template library. However, NSiteMatch significantly outperforms Findsite when the predicted protein shares low structural similarity to the template library. Contrary to the Findsite which relies on identification of templates that have similar topology to the topology of the predicted protein, our method recognizes templates that share local similarity in the binding area and which are not necessarily similar in their overall topology. This allows us to identify similar binding sites across potentially very different protein structures. Our method can accurately, when compared to the current state of the art, find distant functional relationships between proteins from different families, superfamilies, and folds. Although the NSiteMatch targets predictions for a few specific nucleotides, our methodology constitutes a generic platform that could be extended to predict interactions with other small ligands.

## Figures and Tables

**Table 1:** Statistical significance of the differences in distances measured using *D*_*CC*_ between the predicted and the actual location of the binding site measured using Wilcoxon signed-rank test. The “+” indicates that NSiteMatch is significantly better than a method in a given column with *p* < 0.05 and “=” denotes the fact that NSiteMatch and a method in a given column are not significantly different.

Ligand type	SCOP level	MetaPocket	Findsite	Q-SiteFinder
ADP	40%	+	+	+
	Family	+	+	+
	Superfamily	+	+	+
	Fold	+	+	+

ATP	40%	+	+	+
	Family	+	+	+
	Superfamily	+	+	+
	Fold	+	+	+

AMP	40%	+	+	+
	Family	+	+	+
	Superfamily	=	+	=
	Fold	=	+	=

**Table 2:** Comparison of the predictive qualities of the NSiteMatch, MetaPocket, Findsite, and Q-SiteFinder for the prediction of binding residues for ADP, ATP, and AMP. The PRE, REC, and MCC stand for precision, recall, and Matthews Correlation Coefficient, respectively. The NSiteMatch generates a real value for each residue (propensity to bind), which is thresholded to make binary (binding versus nonbinding residue) predictions. The rows annotated as the “NSiteMatch^p^” are based on the thresholds that generate precision values which match the highest precision obtained by the MetaPocket, Findsite, and Q-SiteFinder for a given ligand type; similarly, the “NSiteMatch^r^” rows correspond to thresholds for which the highest value of recall is matched. The matching recall and precision values are shown in italics and the highest MCC values are given in bold font.

Ligand	Method	40%			Family			Superfamily			Fold		
type		PRE	REC	MCC	PRE	REC	MCC	PRE	REC	MCC	PRE	REC	MCC
ADP	NSiteMatch^p^	*0.48*	0.79	0.6	*0.43*	0.68	0.52	*0.43*	0.49	**0.44**	*0.43*	0.48	**0.43**
	NSiteMatch^r^	0.76	*0.53*	**0.62**	0.53	*0.57*	**0.53**	0.37	*0.57*	0.43	0.37	*0.57*	**0.43**
	MetaPocket	0.41	0.13	0.21	*0.43*	0.13	0.22	*0.43*	0.13	0.22	*0.43*	0.13	0.22
	Findsite	*0.48*	0.67	0.55	0.41	0.45	0.42	0.31	0.32	0.3	0.31	0.3	0.3
	Q-SiteFinder	0.29	*0.53*	0.36	0.31	*0.57*	0.39	0.31	*0.57*	0.39	0.31	*0.57*	0.39

ATP	NSiteMatch^p^	*0.49*	0.68	**0.56**	*0.46*	0.54	**0.47**	*0.46*	0.41	0.41	*0.46*	0.41	0.41
	NSiteMatch^r^	0.61	*0.52*	0.54	0.47	*0.51*	**0.47**	0.4	*0.51*	**0.42**	0.4	*0.51*	**0.42**
	MetaPocket	0.47	0.16	0.26	*0.46*	0.14	0.23	*0.46*	0.14	0.23	*0.46*	0.14	0.23
	Findsite	*0.49*	0.52	0.5	0.38	0.43	0.39	0.29	0.34	0.31	0.29	0.34	0.31
	Q-SiteFinder	0.31	*0.52*	0.36	0.29	*0.51*	0.35	0.29	*0.51*	0.35	0.29	*0.51*	0.35

AMP	NSiteMatch^p^	*0.47*	0.62	**0.51**	*0.47*	0.36	0.39	*0.47*	0.3	0.35	*0.47*	0.28	0.34
	NSiteMatch^r^	0.47	*0.62*	**0.51**	0.33	*0.6*	**0.41**	0.29	*0.6*	**0.38**	0.29	*0.6*	**0.38**
	MetaPocket	*0.47*	0.16	0.26	*0.47*	0.15	0.25	*0.47*	0.15	0.25	*0.47*	0.15	0.25
	Findsite	0.44	0.49	0.44	0.31	0.34	0.31	0.29	0.33	0.3	0.29	0.32	0.29
	Q-SiteFinder	0.29	*0.62*	0.39	0.29	*0.6*	0.38	0.29	*0.6*	**0.38**	0.29	*0.6*	**0.38**

**Table 3:** The templates identified by NSiteMatch for the probable cell division inhibitor mind protein. Three templates, namely, phosphoenolpyruvate carboxykinase, UDP-N-Acetylmuramoylalanine-D-Glutamate Ligase, and thermosome alpha subunit, have different topologies but similar binding segments and binding sites to the predicted protein.

Polymer name	PDB code:(chain)	Binding segment							SCOP label
Cell division inhibitor mind protein	1ION:A	G	T	—	G	K	T	T	c.37.1.10
Phosphoenolpyruvate carboxykinase	1K3C:A	G	T	—	G	K	T	T	c.91.1.1
UDP-N-Acetylmuramoylalanine-D-Glutamate ligase	2JFG:A	G	S	N	G	K	S	T	c.72.2.1
Thermosome alpha subunit	1Q3S:A	G	D	—	G	T	T	T	a.129.1.2
